# An Investigation of Polyoxometalate Hybrid Materials as Patternable Dielectrics and Lithographic Resists

**DOI:** 10.3390/ma10111309

**Published:** 2017-11-15

**Authors:** Brandon Hardie, Mark Roll

**Affiliations:** Department of Chemical and Materials Engineering, University of Idaho, Moscow, ID 83844, USA; hard4254@vandals.uidaho.edu

**Keywords:** polyoxometalate, hybrid materials, dielectrics, photolithography

## Abstract

Polyoxometalate (POM) hybrid materials have shown potential as spin-coatable, patternable dielectric thin-films and components for lithographic resists. In particular, the octamolybdate cluster has been shown to possess good spin-coating properties and the patterning capabilities of hybrid octamolybdate thin-films were explored using a combination of broadband UV and electron beam lithography (EBL) techiniques. Dielectric properties of these films were determined by ellipsometry, and octamolybdate clusters were subsequently investigated as negative resists in various blends for potential uses in next-generation photolithography, where contrast, sensitivity, and line edge roughness characteristics were determined. Preliminary evidence for the suppression of the diffusion of photo-generated acids is presented.

## 1. Introduction

Applications utilizing polyoxometalate hybrid material chemistry have continued to make interesting strides over recent years. Ranging from catalysis to nanotechnology and medical science, these novel materials continue to show vast potential in many fields of study [1,2,3,4]. The work outlined within this article focuses primarily on polyoxometalates (POMs) as photo-active thin-films. The initial goal of this project was to assess the capabilities of POMs as photoresist components for next-generation photolithography. Spin-coatable hybrid materials, especially their inorganic component traits, have consistently been sought after for properties such as etch resistance and reduced pattern swelling [5,6]. Also of interest for next-generation photolithography, metal-containing resists more strongly absorb light at extreme ultra-violet (EUV; 13 nm) wavelengths [7]. While several POMs were explored over the duration of this project [8,9], the octamolybdate (Mo_8_) POM cluster, [α-Mo_8_O_26_]^4−^ [10] was directly spin-coatable and patternable under UV exposure, properties that best furthered the primary thin-film patterning objective. Multiple patterning methods were utilized, beginning with high exposure doses from a UV curing lamp, to more controlled, conventional instrumentation such as e-beam lithography (EBL) and a UV mask aligner. Mo_8_ films were found to act as negative-tone resists both alone and in resist blends. 

Lithographic properties such as contrast, sensitivity, and pattern line edge roughness (LER)/resolution were determined through this experimental effort. A combination of profilometry—for physical measurements of film thickness—and electron microscopy—for detailed imaging—were used to characterize these films. An additional topic of interest throughout the progress of this work was patternable high dielectric thin-films. Optical/dielectric properties were obtained by modeling these novel materials using ellipsometry data. The findings within this research have begun some foundations of novel POM applications and may be built upon in the near future to realize new potential applications. 

## 2. Materials and Methods

### 2.1. Octamolybdate Synthesis and Blends

**Figure 1:**
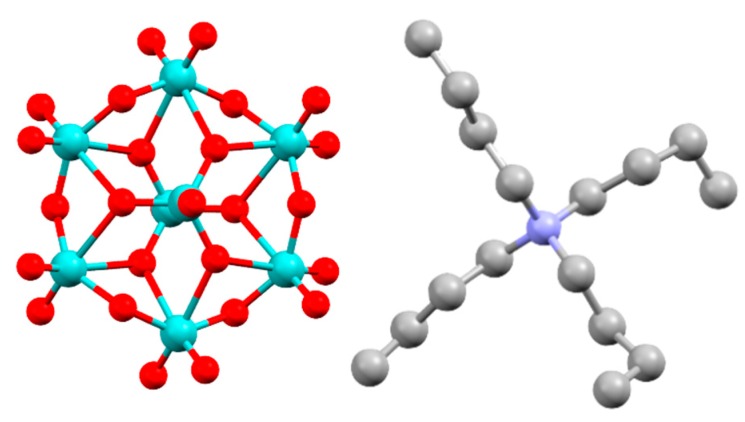
Mo_8_ cluster with tetrabutylammonium cation [11].

The synthesis of the Mo_8_ clusters (Figure 1) was a facile procedure accomplished according to literature [10]. A solution of 4.60 g (14.3 mmol) tetrabutylammonium bromide (Matrix Scientific) in 14 mL water was added dropwise to a solution of 5.00 g (4.04 mmol) ammonium heptamolybdate tetrahydrate (Strem Chemicals) dissolved in ~20 mL water while stirring. A white precipitate was immediately formed and the solution was stirred for 5 min. The product was recovered as a colorless powder, collected via filtration and rinsed with water three times (once with heated water to ensure any excess reactants were removed). Products were confirmed with elemental analysis (Appendix A.1) using an Exeter CE-440 elemental analyzer. In some cases, additional materials were blended in with the Mo_8_ materials to enhance the mechanical and/or lithographic properties of the overall system. These materials included both photoacid generators (PAGs triphenylsulfonium triflate from Synquest Laboratories and *N*-hydroxynaphthalimide triflate from Sigma Aldrich; TPSTF and NHNT, respectively) as well as a molecular tetra-epoxide (tetraphenylolethane glycidyl ether from Sigma Aldrich, 4-EP). Materials were used as received. 

### 2.2. Spin-Coating Optimization

While several conventional casting solvents were employed, it was found that acetonitrile (Fisher Chemical) was the most suitable casting solvent for the Mo_8_ materials, given the poor solubility in other solvents. Materials were coated on Si wafers using a Laurell Technologies WS-650 Series Spin Processor at the following settings: 100 rpm (20 rpm/s) for 10 s, 615 rpm (165 rpm/s) for 35 s, 1000 rpm (500 rpm/s) for 1 min. Physical blends were identified based on their solution compatibility with Mo_8_ in acetonitrile. The same spin settings were used for these blended films. Solutions were typically made at 5% wt. in acetonitrile, with blended systems ranging from 0–100% Mo_8_ ratios (relative to 4-EP). PAG contents in these systems were examined at both 5 and 10% mol based on solids in the system.

### 2.3. Patterning and Development Methods

Several methods of patterning/lithography were used to examine the behavior of Mo_8_ materials. For each technique, Mo_8_ alone and in resist blends acted with negative tone, where unexposed portions of material were removed following a wet development step. Initially, an industrial UV curing lamp (Fusion UV medium pressure mercury lamp; ~200–400 nm wavelength) was implemented for rapid film exposures ranging from estimated doses up to 450 J/cm^2^. Mo_8_ films were also studied using more general, dose-controlled lithography tools. The first of these methods utilized electron beam lithography on a Zeiss Supra 35 VP FEG Scanning Electron Microscope. Films were typically exposed at an accelerating voltage of 20 keV over a dosage range of 5–2500 μC/cm^2^. Exposures using a Quintel Q-4000 UV mask aligner (~365–412 nm) gave feature sizes as low as ~50 μm, given mask availability. All patterning methods were followed by a post exposure bake (PEB) of ~90°C for ~3 min. The development of Mo_8_ only films was accomplished with minor agitation for ~1–3 min in a deionized water bath. For blended Mo_8_ films, a 30-s ethyl acetate bath was used, followed by an isopropyl alcohol rinse prior to the final deionized water bath.

### 2.4. Film Characterization

Optical data of the Mo_8_ films was examined using a JA Woollam VASE ellipsometer. Refractive index (n) and extinction coefficient (k) data were obtained across a wavelength range of 300–1000 nm at a 65° angle of incidence. In addition to ellipsometry, measurements of film thicknesses at varying exposure doses were obtained using a Tencor Alpha Step 200 Profilometer. The primary use of these physical measurements was to construct contrast curves and resist sensitivity data over a range of exposure doses for both EBL and UV octamolybdate blends. 

### 2.5. SEM Imaging

Utilizing a typical accelerating voltage of 5 keV, higher resolution images of Mo_8_ films were obtained. These images allowed a more in-depth look at the film morphology and resolution/line edge roughness of patterned Mo_8_ films, as well as a top-down perspective of photoacid diffusion within the films.

## 3. Results and Discussion

### 3.1. Spin-Coating Optimization

**Figure 2:**
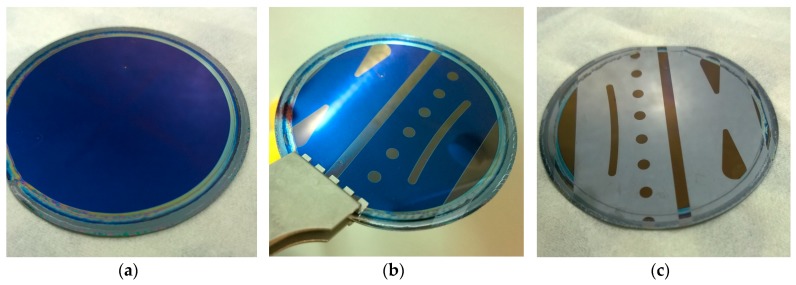
(L to R) Wafer imaging following spin-coating (**a**), post exposure bake (**b**), and final development (**c**)**.**

As previously mentioned, acetonitrile as a casting solvent provided the best uniform film distribution (as seen in Figure 2), while also catering—solubility-wise—to all involved resist components. Given the settings outlined above, a colorless Mo_8_ raw product resulted in a blue color due to thin-film interference, though other colors were observed over a range of spin settings and resist compositions. Octamolybdate POMs by themselves were particularly insoluble in many common casting solvents, including PGMEA and cyclopentanone. While Mo_8_ was also soluble in hot propylene carbonate (<1 wt. %), poor spin-coating results were obtained. While acetonitrile provided sufficient quality films for the purpose of octamolybdate characterization, it is also worth mentioning that some concerns arose from this casting solvent. As a low boiling solvent, some spin-coated wafers did exhibit a vacuum chuck mark which, would almost certainly lead to issues of non-uniformity, especially when working at the nanometer scale. Similar results arose for the blended resist systems. It would therefore be of interest in the near future to examine any potential alternatives to casting solvents in the case that octamolybdates should be applied at a larger scale as a viable resist component.

### 3.2. Patterning and Lithography: Mo_8_ Alone

#### 3.2.1. UV Curing of Mo_8_-Only Resists

To rapidly screen Mo_8_ films for patterning under UV exposure, an industrial UV curing lamp was used. Despite exposure doses orders of a magnitude above that of conventional photolithography (as described in Section 2.3), this method gave some interesting insight on the patterning capabilities of novel POM materials. Figure 2 shows the sequence of the exposure, PEB, and development process. Visually, perhaps the most notable trait following PEB is the color change of the film. In the case of higher exposure doses, it is hypothesized that the organic ligands in the Mo_8_ system decompose under the intense UV light in the ambient atmosphere, resulting in film color change as well as condensing the film thickness. Given the large feature sizes patterned with this method, these films—both before and after exposure—were easily analyzed using ellipsometry. This hypothesis of this film condensing is further examined in Section 3.5, where Mo_8_ optical properties were determined, an ellipsometry model of this film was built, and film thicknesses were determined using this model.

#### 3.2.2. Electron-Beam Lithography (EBL) of Mo_8_-Only Resists

**Figure 3:**
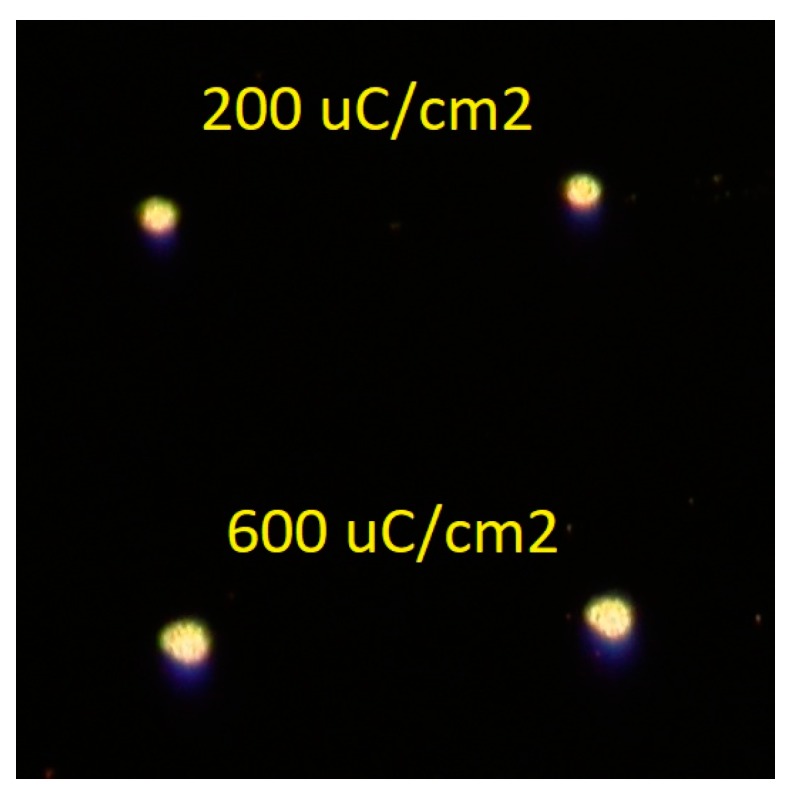
Optical microscope image of Mo_8_-only film patterning quality under electron-beam lithography (EBL).

EBL was implemented as the first look at POM lithographic characterization, considering its much more controlled exposure doses as opposed to the UV curing system. Similar to the UV curing system, early studies of Mo_8_ films developed a pattern without the aid of blended materials. At these much smaller feature sizes (20 μm × 20 μm), however, it was evident that pattern quality for Mo_8_-only films would be a limiting factor moving forward. As shown in Figure 3 below, rather than exhibiting crisp line edges (square raster-scan at a 20-μm feature size), these exposures instead developed with very rounded edges and a rough surface morphology.

### 3.3. Patterning and Lithography: Blended Resists

#### 3.3.1. UV Mask Aligner of Blended Resists

**Figure 4:**
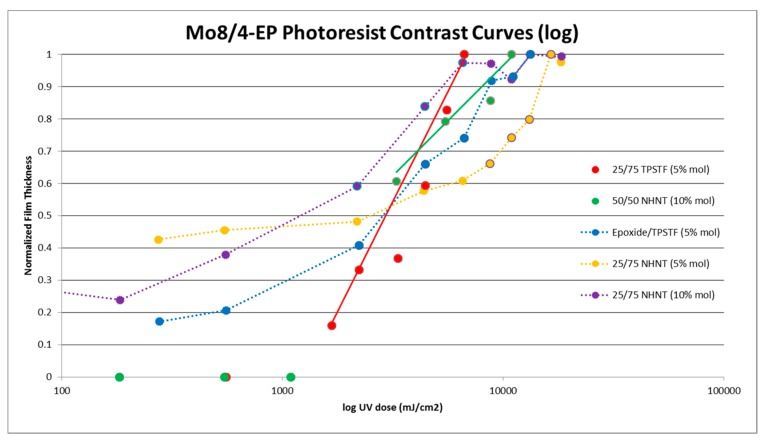
Mo_8_ blend contrast curves using UV exposure (365–412 nm).

**Figure 5:**
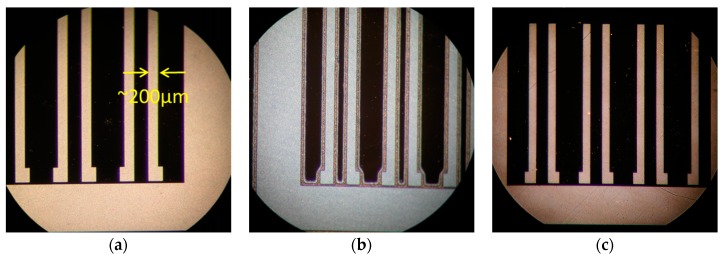
Optical microscope imaging of (L to R) 25:75 TPSTF; triphenylsulfonium triflate (**a**), 25:75 NHNT; *N*-hydroxynaphthalimide triflate (**b**), and 50:50 NHNT (**c**) resist blends.

In an effort to obtain lithographic properties in a more commercially relevant context, a UV mask aligner was utilized next. Unlike the UV curing and EBL patterning methods, however, Mo_8_-only films did not exhibit negative resist characteristics. With this result, it was proposed to use PAGs to create a blended resist material with the octamolybdates. In addition to PAGs, a small molecule tetraepoxide—4-EP—was added for mechanical stability [12] and improving the general surface roughness generally displayed by Mo_8_-only films. Using 4-EP alone provided a standard baseline with which to compare blended films. Several different mixtures of these components were examined at exposure doses ranging up to greater than 10 J/cm^2^. NHNT PAG loads were increased to 10 mol % due to a majority of the mixtures not patterning over this range at 5 mol %. Typical results showed a low contrast—an extended time to reach dose-to-clear—for these mixtures, as seen in Figure 4. Two mixtures—25:75 TPSTF and 50:50 NHNT—as mentioned above, showed “optimal” contrast relative to other blends and were further studied using EBL. The contrast and sensitivity values obtained are as shown in Table 1 for these particular mixtures. Furthermore, imaging taken from optical micrographs compares these “optimal” blends, as well as a 25:75 NHNT mixture to show the impact of increasing the Mo_8_ content (Figure 5).

**Figure 6:**
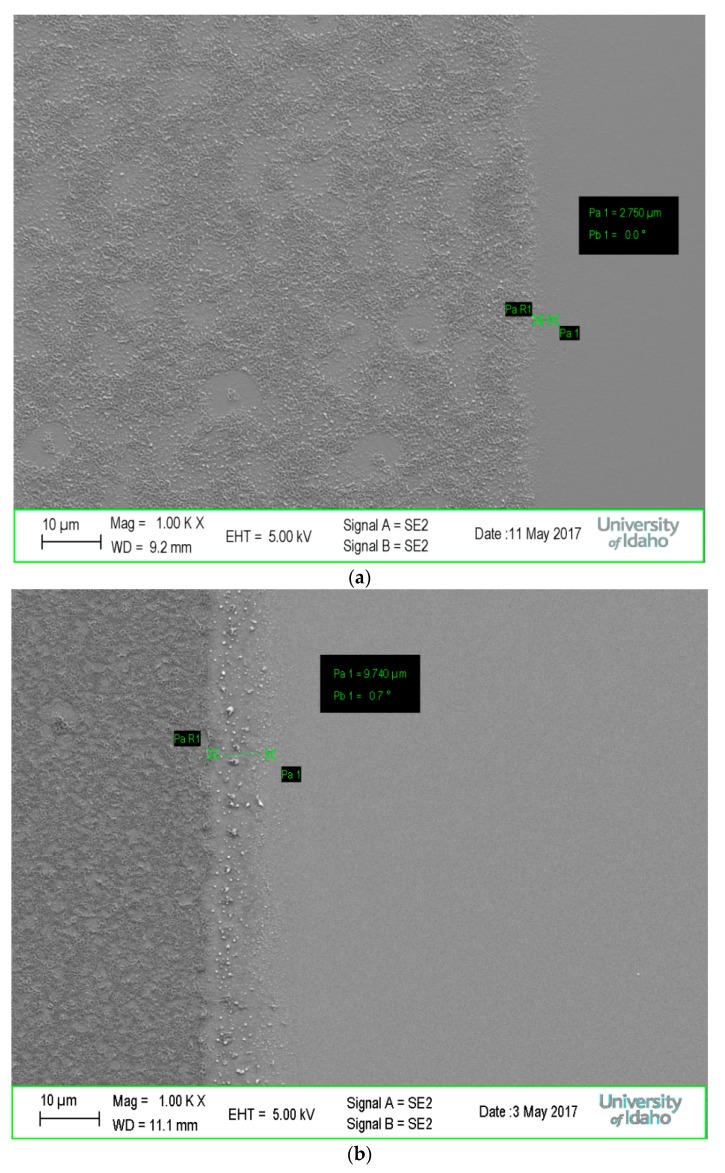
SEM imaging of (**a**) 50:50 NHNT and (**b**) 25:75 TPSTF examining line-edge roughness (LER) UV exposures.

SEM imaging provided a better look at the film morphology and line-edge roughness (LER) of these systems, and Figure 6 shows the comparison of these two systems. It was observed that the 25:75 TPSTF mixture showed an increase in LER of ~10 μm vs. ~3 μm for 50:50 NHNT. Accordingly, it is hypothesized that the higher octamolybdate content continues to reduce LER and effects from photoacid at the micron scale. Also, very rough surfaces were attained through this UV method, leading to more questions regarding the viability of these resists with this lithography technique. Potential improvements of this surface morphology may be traced back to development conditions, such as eliminating the isopropyl alcohol rinse. While film roughness cannot be determined with great detail by the naked eye during development, it would be worthwhile to determine what effects on film roughness, if any, come from varying development conditions.

#### 3.3.2. Electron-Beam Lithography (EBL) of Blended Resists

**Figure 7:**
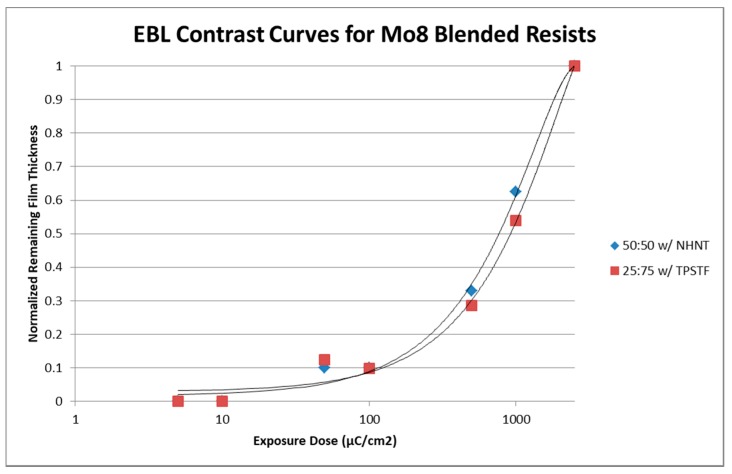
Mo_8_ blend contrast curves using EBL.

With the introduction of blended films, the UV mask aligner was used to screen multiple solutions quickly. The two film blends with “optimal” contrast and sensitivity values (determined from UV characterization) were more extensively examined under EBL. As previously mentioned, these blends consisted of 5% wt. acetonitrile solutions of 50:50 (Mo_8_:4-EP) with 10 mol % NHNT and 5 wt. % 25:75 with 5 mol % TPSTF. These films were exposed at a range of 5–2500 μC/cm^2^ to develop EBL contrast curves of the blended resists. Following the normalization of the film thicknesses as measured via profilometry, it was found that the contrast curves were very similar to one another, regardless of Mo_8_ ratio in the system or which PAG was used (Figure 7). As with the UV exposures, contrast and sensitivity data were obtained for EBL and is presented in Table 2. 

**Figure 8:**
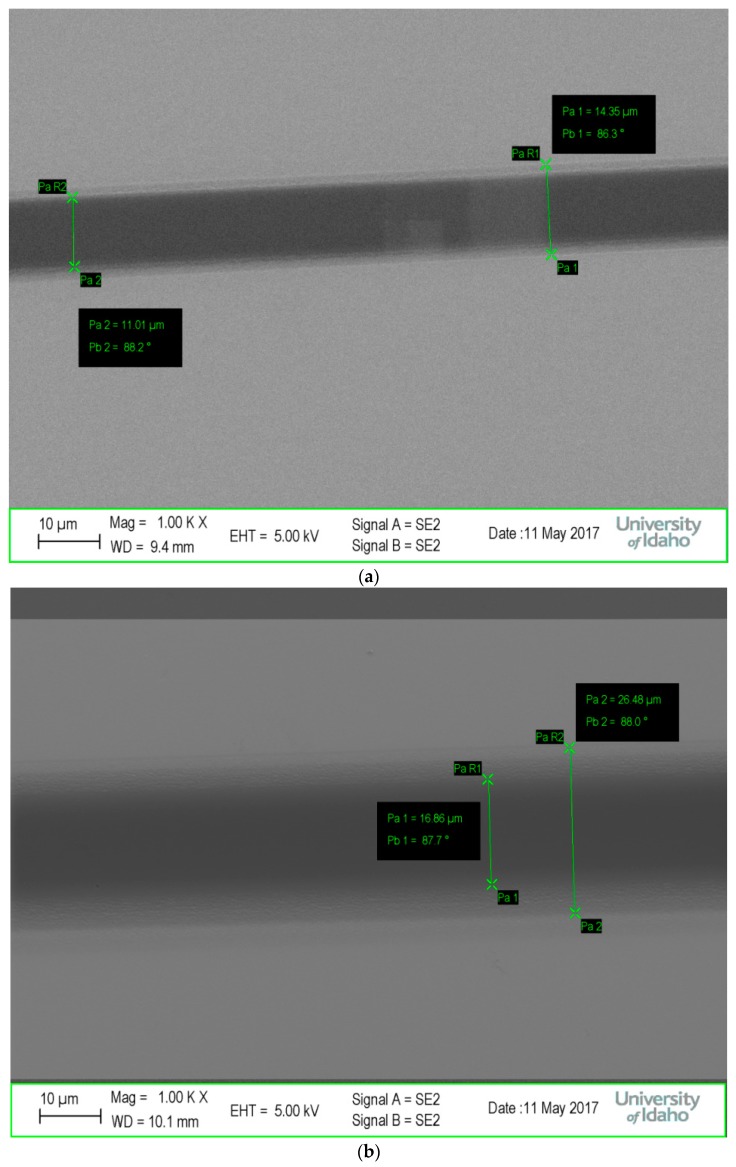
SEM imaging of (**a**) 50:50 NHNT and (**b**) 25:75 TPSTF examining LER.

Given this similarity between the contrast curves of these blends, the exposures were examined in greater detail. In particular, an interesting observation of the PAG diffusion effects on LER was made. Figure 8 shows an SEM image for each of these systems with noticeable swelling of the exposure regions. Of note, the intended width of these exposures was 10 μm (actual measurements as shown in Figure 8). With that in mind, it is immediately observed that the PAGs appear to be a potential cause of significant LER in the overall pattern, especially compared to the previous UV exposures. 

Furthermore, despite having a lower 5% PAG loading, the 25:75 TPSTF mixture shows an increase in pattern width greater than 150%, measuring as wide as ~27 μm. In contrast, the higher PAG loading the 50:50 NHNT mixture, despite evident LER, shows significant improvement, keeping in mind that the 25:75 TPSTF mixture showed optically visible LER. This supports the previous hypothesis that higher Mo_8_ content potentially suppresses LER from photoacid migration. Potentially more effective at cross-linking, the lower Mo_8_ content in the TPSTF-containing resists may also have increased secondary electron scattering in comparison to the 50:50 system, contributing further to LER. EBL also displayed a much-improved surface morphology in comparison to UV exposures. It is important to understand the impact of octamolybdates on these resist blends, as the ability to suppress PAG or secondary electron diffusion by blending POMs with conventional resists—both positive and negative—could have a major impact on novel photoresist chemistry. The ability to precisely manipulate exposure dose in EBL will surely lead to further in-depth studies of LER and general Mo_8_ lithographic properties.

### 3.4. Mechanistic Analysis

Given the potentially complex negative tone action of these resist blends, it is critical to assess the mechanisms that lead to pattern formation. When examining the effects of the added components—PAG and 4-EP—in resist blends, some initial explanations may be proposed. Octamolybdates have been shown to undergo structural changes in the presence of acids forming larger complexes, as for instance [Mo_36_O_112_(H_2_O)_16_]^8−^, or even polymeric chains of coupled octamolybdate clusters [13] during the PEB or the development process, when the solvent swelling of the resist occurs. This would appear to be a suitable explanation for the solubility switch observed, as larger, more highly charged species would be expected to have lower solubilities, though it is critical to acknowledge that such rearrangements in the solid state are much less probable.

This mechanism would also be aided by the presence of PAGs in resist blends. Aside from an apparent improvement in surface morphology, the presence of 4-EP as a negative resist component in these blends would provide additional cross-linking within the system through the epoxide functionality. While the effects of these components in the resist mixtures appears to be at least partially addressed by the mechanisms above, addressing the behavior of Mo_8_-only films may not be as trivial. This is especially notable given that these single component films patterned under EBL (though with poorly defined features) and high-power UV curing, but not under the UV mask aligner. 

The patterning action could be potentially due to the different energies of UV and EBL systems, causing decomposition of the tetrabutylammonium cations and thus a change in solubility for the film. The ellipsometric data does suggest that the refractive index of the films increased, with the most likely explanation being a loss in organic content. Literature studies have shown the capabilities of POMs to oxidize and degrade organic components [14,15]. Further examination (through IR-spectroscopy, etc.) is likely needed to truly understand the mechanism that drives the negative tone patterning of octamolybdate materials. Recent work by Sun and coworkers details a mechanism by which protons and hydroxyl radicals are produced under UV irradiation [16]. Any protons produced would activate the polymerization of the octamolybdate clusters discussed above, and the hydroxyl radicals might attach to the quaternary ammonium cations or engage in other unexpected reactions.

### 3.5. Ellipsometry

**Figure 9:**
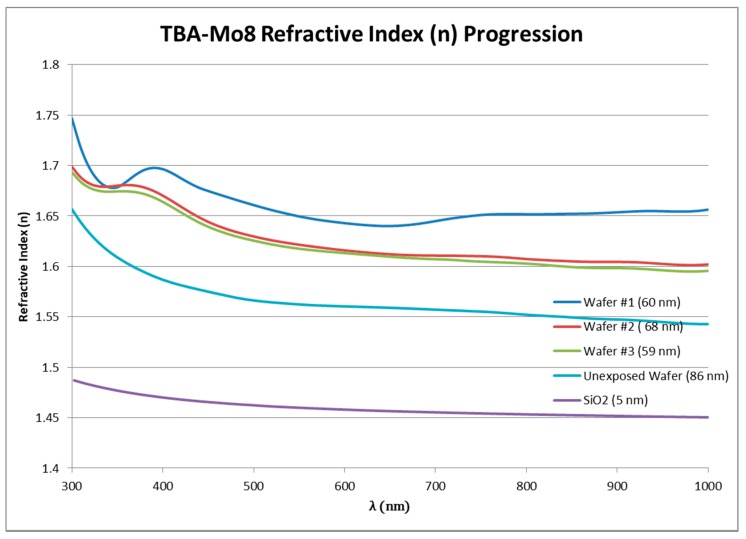
Octamolybdate ellipsometry model building progression of refractive index and calculated thickness.

Ellipsometry studies were exclusively done on octamolybdate films produced by the UV curing system, given that the much smaller feature sizes from the EBL and UV mask aligner patterns were difficult to assess. The goal of the ellipsometry studies was to first develop a model and obtain optical constants n (refractive index) and k (extinction coefficient) for the octamolybdate films. As a novel thin-film material, octamolybdate POMs are relatively unknown in regard to standard ellipsometric models. Modeling began with SiO_2_ as a starting material with a manipulated Cauchy layer. Raw data (Ψ and Δ) from both ellipsometry and reflectivity measurements were eventually developed into a good model fit over several exposure iterations, resulting in the data seen in Figure 9 for the refractive index of several films. The films examined included multiple exposed films (~100–250 J/cm^2^), unexposed, and a SiO_2_ standard film.

**Figure 10:**
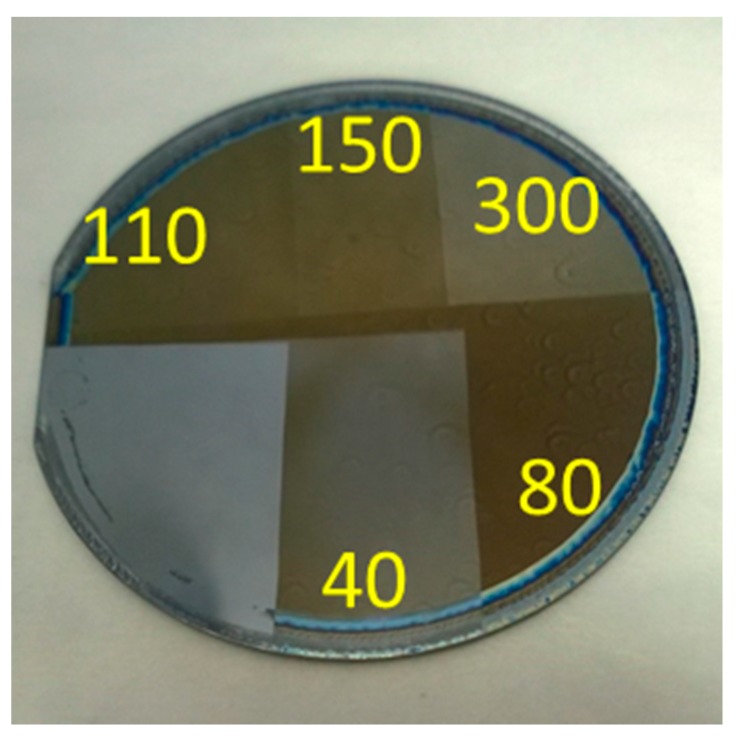
UV cured octamolybdate demonstrating film “burn off” and/or condensation (estimated units in J/cm^2^).

In addition to obtaining optical properties and successfully building a model for octamolybdate films, the indication of film loss/film condensing was observed. As previously mentioned in Section 3.2.1, the potential of organic ligand “burn off” was considered due to the high exposure doses obtained from the UV curing lamp. As with a majority of negative tone photoresists, it would be expected that with increasing exposure doses, film thicknesses would eventually reach a maximum step height and level off. When comparing film thicknesses measured using the newly developed ellipsometry model, it was instead observed that the remaining films began to decrease after reaching a peak. This occurrence was not observed using the more conventional methods of photolithography, and its expected cause was that the energy doses were orders of magnitude larger. Figure 10 gives an example of what was observed in the films as exposure doses were increased. In this image, darker portions of the film show the increasing film thicknesses. Note that UV-curing doses were estimated based on the specifications of the lamp and the exposure area.

### 3.6. Future Work

Studies in the near future will continue to focus on the patterning quality of octamolybdate films. A more in-depth look at PEB will examine the effects of altering both temperature and the PEB time and how this could potentially alter LER and PAG diffusion lengths. While patterning with a negative tone under UV curing and EBL was explored here, Mo_8_ POMs could also be of interest for blends in positive tone resists where PAG and/or secondary electron diffusion remains a concern. While diffusion and LER measurements from a top-down perspective can give good insight into the performance of these films, other techniques can be examined. In particular, a technique similar to that of the group of Kang et al. [17], where a bilayer system is used to characterize PAG diffusion, would be of additional interest in determining PAG migration in the presence of octamolybdate clusters. Extreme ultra violet exposures would also be useful to study how well the Mo_8_ components can improve the absorption of a resist system at the next photolithography node. Not to be limited to Mo_8_ POMs, decavanadate and decatungstate [18] POMs may be of similar interest to the applications explored here with Mo_8_ clusters. Following these in-depth studies on patterning and lithographic capabilities, these films may be further examined for their applications as patternable dielectrics.

## 4. Conclusions

This work investigated POM hybrid materials as patternable dielectrics and resist components, showing them to possess intriguing potential moving forward. While still in its early stages with room for improvement and experimentation with new blends, much data was obtained. It was found that octamolybdate films showed negative tone patterning following UV curing treatment and electron-beam exposure, in addition to more controlled UV doses in resist blends using a mask aligner. Optimal blends were further studied and contrast curves and sensitivity data were determined. In addition to in-depth pattern quality studies, optical data were obtained and an ellipsometric model was built for these novel materials. Film thicknesses were characterized and measured utilizing both physical measurements and an optical model. Further studies will address the suitability of octamolybdate (Mo_8_) POMs not only as photoresist components, but for direct application as patternable dielectrics.

## Figures and Tables

**Table 1:** Contrast and sensitivity data for UV mask aligner exposures.

Resist Components	Sensitivity (mJ/cm^2^)	Contrast
25:75 TPSTF	4000	1.66
50:50 NHNT	2500	1.00

**Table 2:** Contrast and sensitivity data for EBL exposures.

Resists, Mo_8_:4-EP	Sensitivity (μC/cm^2^)	Contrast
25:75 TPSTF (5 mol %)	900	1.4
50:50 (10 mol % NHNT)	750	1.4

## Appendix A

### Appendix A.1. Elemental Analysis Results

Elemental analysis calcd (%): C, 35.7; H, 6.74; N, 2.60

Found: C, 35.8; H, 6.81; N, 2.90
